# Carbon cycling in temperate grassland under elevated temperature

**DOI:** 10.1002/ece3.2210

**Published:** 2016-10-11

**Authors:** Anne B. Jansen‐Willems, Gary J. Lanigan, Ludger Grünhage, Christoph Müller

**Affiliations:** ^1^ Teagasc Johnstown Castle Wexford, Co. Wexford Ireland; ^2^ Department of Experimental Plant Ecology (IFZ) JLU Giessen Heinrich‐Buff‐Ring 26‐32 35390 Giessen Germany; ^3^ School of Biology and Environmental Science University College Dublin Dublin Ireland

**Keywords:** CO_2_, elevated temperature, grassland, heating, isotopes, net ecosystem exchange, respiration

## Abstract

An increase in mean soil surface temperature has been observed over the last century, and it is predicted to further increase in the future. The effect of increased temperature on ecosystem carbon fluxes in a permanent temperate grassland was studied in a long‐term (6 years) field experiment, using multiple temperature increments induced by IR lamps. Ecosystem respiration (R‐eco) and net ecosystem exchange (NEE) were measured and modeled by a modified Lloyd and Taylor model including a soil moisture component for R‐eco (average *R* ^2^ of 0.78) and inclusion of a photosynthetic component based on temperature and radiation for NEE (*R* ^2^ = 0.65). Modeled NEE values ranged between 2.3 and 5.3 kg CO_2_ m^−2^ year^−1^, depending on treatment. An increase of 2 or 3°C led to increased carbon losses, lowering the carbon storage potential by around 4 tonnes of C ha^−1^ year^−1^. The majority of significant NEE differences were found during night‐time compared to daytime. This suggests that during daytime the increased respiration could be offset by an increase in photosynthetic uptake. This was also supported by differences in *δ* ^13^C and *δ* ^18^O, indicating prolonged increased photosynthetic activity associated with the higher temperature treatments. However, this increase in photosynthesis was insufficient to counteract the 24 h increase in respiration, explaining the higher CO_2_ emissions due to elevated temperature.

## Introduction

Global mean surface temperature has increased by 0.6°C in the past century, and it is expected to increase by 1.5–4.5°C when the atmospheric CO_2_ concentration doubles (IPCC, 2013). Climatic warming could, in some regions and climates, stimulate nutrient mineralization and lengthen the growing season, which would increase plant growth and carbon sequestration (Schimel et al. 1996; Menzel and Fabian 1999; Rustad et al. 2001). However, warming could also accelerate biological metabolism, resulting in a greater respiratory release of CO_2_ to the atmosphere via auto‐ and heterotrophic processes (Lloyd and Taylor 1994; Rustad et al. 2001). Increasing the emissions of the climate relevant gas CO_2_ intensifies the greenhouse effect and therefore contributes to enhanced air temperatures (Le Treut et al. 2007). If increased surface temperature results in higher CO_2_ emissions, a positive feedback loop could occur (i.e., rising atmospheric CO_2_ concentrations, and associated temperature, will be self‐reinforced). However, if an increased surface temperature actually leads to a net decrease in CO_2_ emissions, the current increase in atmospheric CO_2_ could be dampened. Due to the spatial variability and the climatic influence on the direction of change in net CO_2_ emissions, the need for small‐scale studies, on the potential consequences of elevated soil and air temperatures across ecosystems at all latitudes and precipitations levels, has been proposed by several scientists (Rustad et al. 2001; Aronson and McNulty 2009).

Soil respiration accounts for approximately two‐thirds of carbon losses from terrestrial ecosystems (Luo 2007), while most studies showed an increase in soil respiration with ecosystem warming (Rustad et al. 2001; Zhou et al. 2007; Lin et al. 2011; Wu et al. 2011). There were also studies that reported no effect of warming on soil respiration (De Boeck et al. 2007). One meta‐analysis showed the effect of warming on soil respiration would be larger in woody ecosystems (Rustad et al. 2001) while in another on no difference in sensitivity to warming between ecosystems dominated by herbaceous and woody vegetation was detected (Wu et al. (2011). Lin et al. (2011) also found a significantly increased seasonal soil respiration from a Tibetan meadow. However, when looking at the average seasonal ecosystem respiration (R‐eco), which includes both plant and soil respiration, no effect of warming was found.

Some research shows respiration values returning back to prewarming values after a couple of years (Luo et al. 2001; Rustad et al. 2001; Melillo et al. 2002). Two possible explanations for this are “substrate depletion,” the depletion of labile soil organic carbon pools, and “thermal adaptation,” a decrease in heterotrophic soil respiration rates per unit microbial biomass in response to a sustained temperature increase (Bradford et al. 2008). Both hypothesis were confirmed by the findings of Bradford et al. (2008).

Warming prompts complex reactions affecting multiple processes such as reaction rates, cell division, and elongation; all of which can influence plant productivity. It can also affect plant productivity indirectly by influencing soil water, nutrient availability, or a lengthening of growing season (Dieleman et al. 2012). The results from previous studies on the effect of warming on aboveground biomass were contradictory (Nijs et al. 1996; Rustad et al. 2001; Kim and Henry 2013; Lu et al. 2013; Zong et al. 2013). Different responses in biomass production under different ecosystem warming trials could be due to the differences in water stress (Zhao and Running 2010). Also, in general net C3 photosynthesis will be low under extreme low temperatures due to low enzymatic activity, and very high temperatures due to decreased stomatal conductance and increased photorespiration and is optimal under intermediate temperatures (Hikosaka et al. 2006; Luo 2007). However, the optimal temperature for photosynthesis can change depending on the growth temperature (Hikosaka et al. 2006). Previous research showed a change in optimal temperature with an increase in growth temperature ranging from 0.1 to 0.35°C °C^−1^ for the temperate species (Cunningham and Read 2002). This change in optimal temperature could dampen the effect of temperature elevations on biomass production. The integrated impacts of increased temperature on plant CO_2_ and water relations over a growing period can also be estimated by the ^13^C and ^18^O signatures of plant material (Scheidegger et al. 2000). The *δ* ^13^C largely depends on the extent of enzymatic fractionation and is directly related to the ratio of intercellular/ambient CO_2_ partial pressure (p_c_/p_a_, Farquhar et al. 1982, 1989). Increased *δ* ^18^O on the other hand indicates higher rates of transpiration as ^16^O is preferentially evaporated (Ehleringer and Sandquist 2006).

Only little is known on the effect of ecosystem warming on NEE. The results of previous studies were, as already suggested by the different responses of ecosystem warming on R‐eco and biomass production, contradicting (De Boeck et al. 2007; Xia et al. 2009; Wu et al. 2011; Kim and Henry 2013; Peng et al. 2014). A possible explanation for the different effects of warming on NEE is that photosynthesis tends to reach an optimum at lower temperatures than respiration does (De Boeck et al. 2007; Luo 2007). Also the inequality of water stress among the studies could explain the different responses in NEE. First of all, water stress was suggested to be an explanation for different response in biomass productions. And furthermore, soil moisture status also influences soil biota. Raich and Potter (1995) described the influence of moisture on soil biota in three phases: (1) when moisture levels are low an increase in moisture will lead to an increase in metabolic activity; (2) when there is sufficient soil moisture a change in soil moisture will have little effect on CO_2_ emissions; and (3) very high moisture levels will affect the diffusion of gases in the soil and inhibit aerobic respiration.

Previous meta‐analyses of experimental temperature manipulations included more than 200 experimental sites (Rustad et al. 2001; Wu et al. 2011; Lu et al. 2013; Zhang et al. 2015). However, there is a paucity of information on the effect of warming on CO_2_ emissions from continental European grassland ecosystems. In addition to the lack of results from long‐term field experiments, previous research generally only focussed on one temperature elevation (Peterjohn et al. 1993, 1994; Harte et al. 1995; Nijs et al. 1996; Alward et al. 1999; Grime et al. 2000; Luo et al. 2001; Melillo et al. 2002; Eliasson et al. 2005; Xia et al. 2009; Lin et al. 2011). Soil and plants can respond differently to different temperature elevations (Luo 2007). This study on grassland will therefore look at the effect of different temperature elevations. The objectives of this study were as follows: (1) to examine the effect of 6 years of different elevated temperatures on NEE and R‐eco and sward biomass productivity; and (2) to integrate measurements of NEE and R‐eco with empirical models to produce long‐term estimates.

## Materials and Methods

### Site description

The experiment was established on a permanent grassland of the “Environmental Monitoring and Climate Impact Research Station Linden” in Germany. This research station is located at 50°31.6′N and 8°41.7′E at an elevation of 172 m above sea level. The site had not been ploughed for at least 100 years. During the last decades, it had been managed as a meadow with two cuts per year and fertilized with 50–80 kg N ha^−1^ year^−1^. Since 1995, the amount of fertilizer had been reduced to 40 kg N ha^−1^ year^−1^. The mean annual temperature and precipitation were 9.5°C and 560 mm (observation period: 1995–2014), respectively. The soil is a stagno‐fluvic gleysol on loamy–sandy sediments over clay (FAO classification). The vegetation is characterized as an *Arrhenatheretum elatioris* Br.Bl. *Filipendula ulmaria* subcommunity (Rodwell 1992) and is dominated by 12 grass species, two legumes, and 15 nonleguminous herbs.

### Field treatment

For this experiment, a 100 m^2^ site was divided into 16 equally sized plots, four rows of four plots. Each plot was under the influence of one IR lamp (except for the control), elevating the temperature of both the plants and the soil. An area of 318 cm^2^ in the center of each plot, directly underneath the IR lamp, was used for all measurements between 2008 and 2014. Although this area is small, it would be inappropriate to take measurements outside this area, as the soil further away from the IR lamp would not receive the same treatment. Each plot was assigned to one of four treatments according to a Latin square (each treatment occurred once in each row and each column). The areas of measurement were 2.5 m apart to prevent any contamination between treatments. The four treatments were based on the increase of soil temperature measured at 5 cm depth. The temperature at this depth was elevated by 0, 1 (mean 0.8 standard error 0.02), 2 (mean 1.9 standard error 0.03), or 3 (mean 2.6 standard error 0.03) °C. These temperature treatments are hereafter referred to as T_control_, T_1_, T_2_, and T_3_, respectively. Plot 1 through 16 were respectively assigned to the following treatments: T_control_, T_1_, T_2_, T_3_, T_2_, T_3_, T_control_, T_1_, T_1_, T_control_, T_3_, T_2_, T_3_, T_2_, T_1,_ and T_control_.

The temperature of the plots was elevated using Edison screwbase ceramic infrared heaters of 230V and 250W with reflector and E27 ceramic lamp holder (Friedrich Freek GmbH, Menden, Germany). The lamps were connected to three metal bars to ensure that strong winds would not cause a displacement. A metal plate above the lamps functioned as a rain protector. The IR heaters were placed at different heights above the ground to create different temperature elevations. For treatment T_1_, T_2,_ and T_3,_ the IR heaters were placed respectively at 125 cm, 80 cm, and 55 cm above the ground. There was no infrared heater above the control treatment; however, a “rain protector” plate was installed at 55 cm to ensure comparability to the other treatments. Warming commenced on January 24, 2008.

### Carbon dioxide measurements

CO_2_ measurements were taken between November 2007 and December 2010 and then from March 2013 until February 2014. On November 7, 2007 a LI‐COR collar had been inserted in the middle of each plot. Between 2007 and 2010, R‐eco was measured using a LI‐COR LI‐8100A Analyzer Control Unit (LI‐COR Biosciences, Lincoln, NE) connected to a 20 cm survey chamber (LI‐8100‐103). This is a hand‐held system and could therefore easily be used to measure CO_2_ fluxes at 16 different plots. During this period, measurements were taken on 147 days. On each of the days, CO_2_ fluxes were measured once at each plot, except for 2 days were fluxes were measured three or four times on each plot. In 2013–2014, measurements were made using both dark and clear long‐term chambers. Dark chambers were used to measure R‐eco while clear chambers were used to quantify fluxes of NEE (the net flux of gross primary productivity and R‐eco). CO_2_ fluxes (R‐eco and NEE) were measured using a LI‐COR 8150 multiplexer with LI‐COR LI‐8100A Analyzer Control Unit (LI‐COR Biosciences) and a long‐term chamber (LI‐8100‐104) on each plot. The multiplexor allowed soil CO_2_ flux measurements to be measured automatically in the field for the duration of the experiment. Before each flux measurement, the chamber was vented for 45–50 sec. After that, CO_2_ measurement occurred for 105 or 120 sec (depending on the expected temperatures during measurement period). Throughout each campaign the flux was, for at least seven consecutive days, measured once every hour on each plot. During most campaigns, 16 dark chambers were used, except for three campaigns, where 3–4 dark chambers were replaced by clear chambers. The duration of each measurement campaign, if and where clear chambers were used, and the order of measurement can be found in Table 1. During each measurement, the CO_2_ concentration in the chamber was monitored every second. The CO_2_ flux was calculated using only the measurements taken after the deadband, which is the time between closing of the chamber and reaching a steady mix of gases in the chamber. The deadband was normally set to 30 sec. However, if the *R* ^2^ of the flux was below 0.90 and a visual inspection of the CO_2_‐concentrations over time indicated a steady mix of gases had not yet been reached, the deadband of the measurement was increased. Changing the deadband led to a newly calculated flux and *R* ^2^. Flux measurements with an *R* ^2^ below 0.90 were discarded during data analysis.

**Table 1:** CO_2_ flux measurements took place between 2007 and 2014. Dark chambers were used to measure R‐eco and clear chamber for NEE. Campaign length is the time span in which measurements took place every hour. # is the number of measurements taken. Order is the plot order in which the measurements were taken. In April, July, and October, clear chambers were moved every 24 h. The bold number is the day of the campaign followed by the plots with the clear chambers

Date	Chamber type	Campaign length	#	Order	Clear chamber position
Nov–Dec 2007^a^	Dark		240	Consecutive	
2008–2010	Dark		2352	Consecutive	
Mar 2013^b^	Dark	21 days			
April 2013	Dark/Clear	8 days			**1:** 13,14,15,16; **2:** 1,3,4; **3:** 9,10,11; **4:** 4,11,15; **5:** 3,4,14; **6:** 2,10,16; **7:** 5,6,8; **8:** 5,11,12
June 2013	Dark	7 days	Dark: 31,344 (1523 discarded^c^)		
July 2013	Dark/Clear	8 days		Altered between treatments, in random order. 5, 13, 8, 16, 3, 11, 15, 7, 14, 4, 9, 1, 12, 6, 2	**1:** 1,2,3,4; **2:** 13,14,15,16; **3:** 9,10,11,12; **4:** 3,5,8,15; **5:** 1,4,6,14; **6:** 2,9,10,16; **7:** 5,6,7,8; **8:** 7,11,12,13
Aug 2013	Dark	7 days	Clear: 3068 (275 discarded^c^)		
Sept 2013	Dark	10 days			
Oct 2013	Dark/Clear	8 days			**1:** 13,14,15,16; **2:** 1,2,3,4; **3:** 9,10,11,12; **4:** 3,5,8,15; **5:** 1,4,6,16; **6:** 2,9,12,14; **7:** 5,7,6,8; **8:** 7,10,11,13
Nov 2013	Dark	7 days			
Dec 2013	Dark	7 days			
Feb 2014	Dark	8 days			

a Pretreatment measurements.

b First 6 days no chamber on plot 4 and 10, thereafter no chamber on plot 9 and 14.

c Measurements discarded because of technical difficulty or *R* ^2^ below 0.9.

### Measurements of soil and biomass

Soil samples were taken to a depth of 7.5 cm in November 2007, before the start of the experiment, and to a depth of 15 cm on May 12, 2014. During the soil sampling in 2007, 40 samples were taken in the middle of each of the four edges for each plot. In 2014, the soil samples were taken shortly after the lamps were turned off (May 12, 2014), from within the LI‐COR collar. The samples were divided into two depths: 0–7.5 cm and 7.5–15 cm. Samples were taken with a standard ring of 250 cm^3^, and fresh weight was determined. Root biomass was determined for both depths. Subsamples were oven‐dried at 105°C. Based on the water content and the volume, the dry bulk density was calculated. Some of the subsamples for the top layer were ground and analyzed by a CNH Macro Elemental Analyzer (Hanau, Germany) for carbon and nitrogen content. Another part of the subsample was heated to 375°C for 24 h to determine the organic matter content (loss on ignition).

On the day before the start of each CO_2_ flux measurement campaign in 2013–2014, except for the September campaign, grass was cut to 5 cm. All samples were dried for 24 h at 105°C and weighed to determine the dry biomass yield. After the IR lamps were turned off, all aboveground biomass was taken and separated into different species. These plant samples were dried at 70°C for 48 h and milled to a fine powder. The isotope ratios ^13^C/^12^C and ^18^O/^16^O in organic leaf matter were determined with a continuous flow isotope ratio mass spectrometer (DELTA‐S; Finnigan MAT, Bremen, Germany). Two elemental analyzers were connected to the mass spectrometer (EA‐1110 for ^13^C/^12^C analysis and EA‐1108 for ^18^O/^16^O analysis, Carlo Erba, Milan, Italy) via an open‐split interface. The plant samples were put in tin capsules, 4.5–5 mg for the ^13^C/^12^C determination and combusted to CO_2_. In terms of ^18^O/^16^O analysis, 1.2‐ to 1.5 mg samples were used and pyrolized to CO for the determination of ^18^O/^16^O. The isotopic values were expressed in delta notation (per mille), relative to V‐PDB for carbon, and V‐SMOW for oxygen.

### Measurements of model covariates

During the 2013–2014 period, soil temperature of each plot was measured using a LI‐COR 8150‐203 temperature probe. The probe was connected to the chamber, and measurements occurred while the chamber was active. Four permanently installed Pt‐100 sensors (Imko, Ettlingen, Germany), on adjacent sites, were used for constant temperature measurements. Temperature in the plot, for modeling yearly fluxes, has been calculated by adding the seasonal average temperature increment, following Table 2, to the temperature measured by the permanently installed probes. Soil water content was measured once a day (except for the weekend) with a TDR sensor. Global radiation data were measured by a weather station at the field site at half hourly averages (http://www.hlug.de/?id=7122&station=1005).

**Table 2:** Treatment temperature elevation (°C). Elevation based on measurements between March 2013 and February 2014. Winter was considered to start December 1, Spring on March 1, Summer on June 1, and Autumn on Sept 1. Day would be between 7:00 and 18:59 and night between 19:00 and 6:59

Season	Day/Night	T_1_	T_2_	T_3_
Winter	Day	0.6 (0.02)	1.2 (0.04)	1.7 (0.06)
	Night	0.5 (0.02)	1.1 (0.04)	1.8 (0.06)
Spring	Day	1.1 (0.05)	2.2 (0.08)	3.2 (0.09)
	Night	0.6 (0.03)	1.2 (0.04)	2.1 (0.06)
Summer	Day	1.5 (0.08)	3.6 (0.14)	3.6 (0.11)
	Night	0.6 (0.03)	1.3 (0.06)	2.4 (0.04)
Autumn	Day	0.9 (0.05)	2.2 (0.07)	2.6 (0.05)
	Night	0.6 (0.03)	1.6 (0.03)	2.4 (0.04)

Between brackets is the standard error of the mean.

### Statistical analyses and calculations

#### Carbon dioxide measurements

All flux measurements were viewed and exported using the FV8100 file viewer software (LI‐COR Biosciences). This was also used for recalculation of fluxes if the deadband had to be changed. All data were prepared for analyses using Microsoft Excel 2013 (Redmond, WA). Statistics and modeling were carried out using IBM SPSS statistics version 22 (Chicago, IL) and SAS version 9.3 (Cary, NC) for the GLIMMIX and MIXED procedure. Differences were considered significant when *P* < 0.05.

The MIXED procedure was used to analyze the equality of plots preheating (square‐root‐transformed data), the 2008–2010 data (log10(1/flux) transformed), and the 2013–2014 log‐transformed daily averages of R‐eco measurements. A Tukey–Kramer adjustment was used to correct for multiplicity effects in pairwise comparisons. Residual checks were made to ensure that the assumptions of the analysis were met. Some outliers were determined after inspection of boxplot (four for preheating, two for 2008–2010 data). Inclusion/exclusion of outliers did not lead to different results. The *P* values given are from the tests with inclusion of the outliers.

Log‐transformed daily averages of the NEE measurements for the months April, July, and October were analyzed using the GLIMMIX (SAS Institute, 2011) procedure. This procedure extends the generalized linear model and incorporates correlations among responses (Schabenberger 2005). A spatial correlation structure on time interval was used to model repeated measurements on plots, and the observation period (day) was a random factor to allow for incompleteness of the factor. A Tukey adjustment was used to correct for multiplicity effects in pairwise comparisons. Residual checks were made to ensure that the assumptions of the analysis were met. Correlations between NEE and covariates were tested using the Spearman's correlation.

#### Modeling ecosystem respiration and net ecosystem exchange

Hourly treatment averages from the frequent 2013 and 2014 measurements were used for modeling R‐eco and NEE. R‐eco was modeled using equation (1), and NEE was modeled using equation (2). In these equations, soil temp corresponds to half hourly average soil temperature at 5 cm depth in Kelvin, soil moisture is in %, and radiation is the average half hourly global radiation in W m^−2^. The model for R‐eco is a variation of the Lloyd and Taylor model (Lloyd and Taylor 1994, eq. 10). The Lloyd and Taylor model does not include optimum and maximum temperatures; however, those lie outside of the range observed in soil. This model can therefore adequately describe the effect of temperature on R‐eco (Kirschbaum 2000). The first part of the equation $\left(\alpha_{1}\cdot e^{-\frac{308.56}{\text{temp}-\alpha_{2}}}\right)$ is the Lloyd and Taylor model. The second part of the equation $\left(e^{-\frac{{\left(\text{soilmoisture}-\alpha_{3}\right)}^{2}}{{\alpha_{4}}^{2}}}\right)$ has the shape of a bell curve and gives a value between 0 and 1. It therefore decreases the R‐eco under very low and high soil moisture conditions. This effect of soil moisture on soil biota has been shown before (Raich and Potter 1995), and furthermore, the data from the current experiment suggest a substantial decrease in R‐eco during drought. The model has been tested by selecting a random 30% of the data for parameterization and the remaining 70% for validation. After validation of the model, the parameters have been reparameterized based on the full data set; these parameters are reported. NEE is modeled based on R‐eco minus a photosynthetic component. The photosynthetic component is determined by radiation and temperature. The R‐eco component is equation (1), and the parameters are based on the R‐eco data and not the NEE data as the R‐eco data set was ten times larger and collected through the entire year. Parameters *β*_1_–*β*_3_ were optimized using parameters for R‐eco based on R‐eco measurements in the months with NEE measurements (March, April, July, and October) to minimize the error induced by an error in the R‐eco estimate. The *R* ^2^ for R‐eco estimates in these months was 0.89 (SD 0.01).

(1) $$\text{R‐eco}=\alpha_{1}\cdot e^{-\frac{308.56}{\text{temp}-\alpha_{2}}}\cdot e^{-\frac{{\left(\text{soilmoisture}-\alpha_{3}\right)}^{2}}{{\alpha_{4}}^{2}}}$$

(2) $$\text{NEE}=\text{R‐eco}-\beta_{1}\cdot \text{radiation}\cdot e^{-\frac{{\left(\text{temp}-\beta_{2}\right)}^{2}}{{\beta_{3}}^{2}}}$$

Daily R‐eco and NEE were calculated based on linear interpolation between half hourly modeled R‐eco/NEE rates. Yearly and monthly total R‐eco and NEE were calculated via summation of the daily rates.

#### Biomass and soil samples

Monthly biomass yields were compared using a mixed ANOVA. The data were transformed using log10(yield/10 + 1) so assumptions of no outliers, a normal distribution, and homogeneity of variances were not violated.

Soil carbon, C/N ratio, bulk density, organic matter content, root biomass, and root‐shoot ratio were compared using the nonparametric Kruskal–Wallis test because during the 2014 soil sampling one sample per plot was taken, resulting in only four measurements per treatment. The samples from 2007 and 2014 were not analyzed with a paired comparison as the sample locations were not the same in 2007 as in 2014. Soil samples from 2007 were compared using an independent sample *t*‐test, as no outliers were observed in the boxplot, the data were distributed normally and there was homogeneity of variances and the sample size was large enough.

## Results

### Change in soil temperature

Soil temperature was altered using IR heaters. The average increase in soil temperature at 5 cm depth, for T_1_, T_2,_ and T_3_, respectively, was 0.8 (SE 0.02), 1.9 (SE 0.03), and 2.6 (SE 0.03) °C. However, the changes differed among seasons (Table 2). The increments were lowest during the winter and the highest during the summer. During winter and autumn, the increments in temperature did not differ much between day and night. During spring and summer, the temperature increments measured as 5 cm depth were higher during the day than during the night.

### Measurement of plot equality

Before the heating trial started, 15 R‐eco measurements per plot were taken. Due to relatively high fluxes from plot 16 and low fluxes from plot 4, significant differences between the 16 plots were found (*P* < 0.01). In the first 3 years after the start of the experiment, no significant differences were found between the four replicates within each treatment.

### The effect of ecosystem warming on ecosystem respiration

#### Short (up to 3 years)‐ and long (5–6 years of ecosystem warming)‐term effects

For the first 3 years after experiment setup, ecosystem respiration differed significantly between the four treatments (*P* < 0.001). Ecosystem was in the order: T_control_ < T_1_ < T_2_ < T_3_ (Table 3). In the final year of the experiment, ecosystem respiration was in the following order: T_1_ < T_control_ < T_3_ < T_2_ (Table 3). Ecosystem respiration under T_2_ and T_3_ did not differ significantly. Highest fluxes were found in the summer, and the lowest fluxes were found in the winter.

**Table 3:** Median of ecosystem respiration (R‐eco) and net ecosystem exchange in 10^−4^ g CO_2_ sec^−1^ m^−2^. Short‐term R‐eco results (2007–2010) are averages instead of medians. Long‐term R‐eco results are based on 2013–2014 measurements

	T_control_	T_1_	T_2_	T_3_
R‐eco short term	2.42^a**b**^	2.62^c(d)^	2.93^a(d)^	3.06^**b**c^
R‐eco long term	1.59^e(f)^	1.19^eg^	2.25^e^	2.05^(f)g^
NEE spring	−0.26	−0.18	−0.44^h^	0.22^h^
NEE summer	3.17^i^	1.85^**jk**^	6.42^i**j**^	4.84^**k**^
NEE autumn	0.88^(l)^	0.92^(m)^	2.16^(l)(m)^	1.54

Significant differences are shown using letters (*P* < 0.05) and bold letters (*P* < 0.01). Tendencies are shown with letters between brackets (*P* < 0.1).

#### Modeling ecosystem respiration

**Figure 1:**
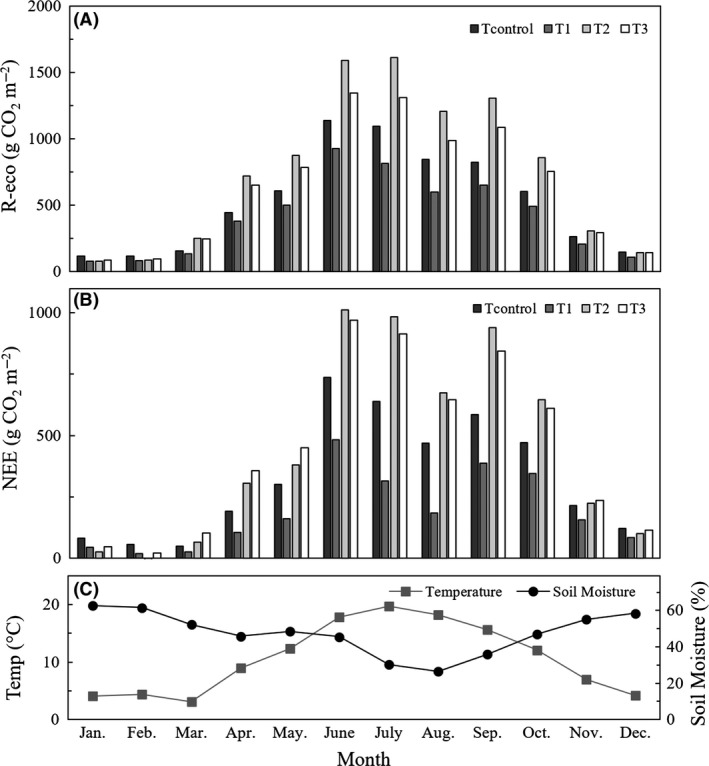
Monthly modeled CO_2_ fluxes from March 1, 2013 to February 28, 2014. Where (A) is ecosystem respiration (R‐eco), based on half hourly soil temperature data (5 cm) and daily soil moisture data according to equation (1) with the parameters given in Table 4; (B) is net ecosystem exchange of CO_2_ (NEE), based on half hourly soil temperature (5 cm) and global radiation data, and daily soil moisture data according to equation (2) with the parameters given in Table 4; and (C) is the monthly average temperature and soil moisture.

The model used for predicting ecosystem respiration has been validated using 30% of the data for parameterization and the other 70% for validation. For all treatments, the *R* ^2^, of both the parameterization set and the validation set, was within 0.02 of the *R* ^2^ reported for the full data set. The RMSE ranged between 0.66 × 10^−4^ and 1.3 × 10^−4^ for the full data set. The RMSE, of both the parameterization set and the validation set, was within 0.04 × 10^−4^ of the RMSE of full data set. The results reported in this section are based on the full data set. Regression analyses between R‐eco rates (average per treatment of measurements taken within an hour) and soil temperature according to equation (1) resulted in an average *R* ^2^ of 0.78 (SD 0.03). Excluding the moisture effect in the model resulted in an average *R* ^2^ of 0.68 (SD 0.04). Table 4 shows the parameters for the model as described by equation (1). Total R‐eco between March 1, 2013 and February 28, 2014 was 6.35 kg CO_2_ m^−2^ for the control treatment compared to 4.97 kg CO_2_ m^−2^ for T_1_, 9.03 kg CO_2_ m^−2^ for T_2_ and 7.78 kg CO_2_ m^−2^ for T_3_. Figure 1A shows monthly sums of the modeled R‐eco for the different treatments. Modeled fluxes were generally in agreement with the statistics performed on the measured data. Highest fluxes were found in the summer and lowest in the winter. Through the entire year highest fluxes were found under T_2_ and T_3_ and lowest under T_1_.

**Table 4:** Parameter including 95% confidence interval for modeling ecosystem respiration (R‐eco) and net ecosystem exchange (NEE) according to the following equations: $\text{R‐eco}=\alpha_{1}\cdot e^{-\frac{308.56}{\text{temp}-\alpha_{2}}}\cdot e^{-\frac{{\left(\text{soilmoisture}-\alpha_{3}\right)}^{2}}{{\alpha_{4}}^{2}}}$ and $\text{NEE}=\text{R‐eco}-\beta_{1}\cdot \text{radiation}\cdot e^{-\frac{{\left(\text{temp}-\beta_{2}\right)}^{2}}{{\beta_{3}}^{2}}}$. Where R‐eco and NEE are in g CO_2_ sec^−1^ m^−2^, soil temperature is the temperature at 5 cm depth in Kelvin, soil moisture is in %, and radiation is in W m^−2^

	T_control_ (R‐eco: *R* ^2^ = 0.80) (NEE: *R* ^2^ = 0.61)			T_1_ (R‐eco: *R* ^2^ = 0.79) (NEE: *R* ^2^ = 0.58)			T_2_ (R‐eco: *R* ^2^ = 0.74) (NEE: *R* ^2^ = 0.76)			T_3_ (R‐eco: *R* ^2^ = 0.81) (NEE: *R* ^2^ = 0.66)		
*α*_1_	0.0975	**0.1080**	0.1184	0.0674	**0.0743**	0.0811	0.077	**0.0861**	0.0951	0.0611	**0.0668**	0.0725
*α*_2_	233.7	**234.6**	235.6	232.5	**233.5**	234.6	227.3	**228.7**	230.1	227.4	**228.5**	229.7
*α*_3_	42.1	**42.8**	43.6	42.0	**42.5**	43.0	38.9	**39.2**	39.5	39.5	**39.8**	40.1
*α*_4_	23.8	**25.3**	26.8	21.8	**20.9**	20.1	16.8	**17.4**	18	18.8	**18.2**	17.7
*β*_1_ × 10^7^	5.055	**6.061**	7.066	6.177	**6.747**	7.317	8.426	**9.546**	10.667	5.291	**6.340**	7.390
*β*_2_	285.4	**288**	290.7	288.1	**289.4**	290.7	288.2	**289.1**	290	287.3	**288.5**	289.7
*β*_3_	8.3	**14.4**	20.6	11.6	**14.1**	16.6	11.6	**14.2**	16.8	10.2	**14.2**	18.2

Lower and upper limit of the 95% confidence interval are given respectively left and right of the parameter estimation. Parameter estimation is given in bold. NEE parameters are optimized using parameters for R‐eco based on R‐eco measurements in the months with NEE measurements (*R* ^2^ = 0.89) to minimize the error induced by an error in the R‐eco estimate.

### The effect of ecosystem warming on net ecosystem exchange

#### Long‐term effects (5–6 years)

Similar results were found for the effect of elevated temperature on NEE as for R‐eco. However, not as many of the differences were significant. Positive NEE values indicate a net CO_2_ released from the ecosystem (i.e., more CO_2_ is lost via respiration than it is taken up by photosynthesis). As there was a significant treatment × month effect (*P* = 0.006), the results are presented on a per month basis (Table 3).

When separating the fluxes during day‐ and night‐time, of the 18 comparisons (four treatments in each of three seasons) only two were found to be significantly different during the day, compared to eight during the night.

#### Modeling net ecosystem exchange

Regression analyses of NEE (average per treatment of measurements taken within an hour) and both soil temperature and global radiation according to equation (2) explained on average 65% of the variation in NEE (SD 0.07). The *R* ^2^, of both the parameterization set and the validation set, was within 0.04 of the *R* ^2^ reported for the full data set, for all treatments except T_3_. For T_3_, the *R* ^2^ was within 0.07 of the *R* ^2^ for the full data set. The RMSE for the full data set ranged between 0.93 × 10^−4^ and 1.6 × 10^−4^. The RMSE, of both the parameterization set and the validation set, was within 0.10 × 10^−4^ of the RMSE of the full data set. The parameter estimations from the regression analyses of the full data set, shown in Table 4, were used to model yearly NEE. NEE between March 1, 2013 and February 28, 2014 was 3.91 kg CO_2_ m^−2^ for the control treatment compared to 2.30 kg CO_2_ m^−2^ for T_1_, 5.34 kg CO_2_ m^−2^ for T_2_ and 5.31 kg CO_2_ m^−2^ for T_3_. Figure 1B presents monthly cumulatives of the modeled NEE for the different treatments. As for the modeled R‐eco, modeled NEE was in agreement with the statistics performed on the measured data. Again, highest NEE fluxes were found in the summer and lowest in the winter (Fig. 1B).

### The effect of ecosystem warming on biomass production

The total biomass yield between the 28th of February 2013 till the 5th of February 2014 was significantly different between the four treatments (*P* = 0.015). It was in the following order: T_1_ < T_3_ < T_control_ < T_2_, where the biomass yields were, respectively, 4.1 (SD 1.9), 4.4 (SD 2.4), 4.9 (SD 1.4), and 9.5 (SD 3.2) tonnes of dry matter per hectare. When looking at the pairwise comparisons only T_2_ had a significantly higher biomass yield than T_1_ (*P* = 0.016) and tended to have a higher biomass than T_control_ (*P* = 0.067). Based on sampling in June 2014, no overall treatment differences were found in the amount of shallow and deep roots, the fraction of deep roots, and the root‐shoot ratio.

**Figure 2:**
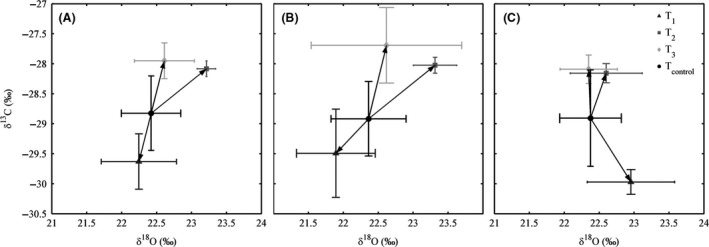
Carbon and oxygen isotope ratios, based on leaf material from the May 2014 harvest. Where (A) is the average of the plots, (B) the average for grasses, and (C) for herbs. Error bars are the standard deviation.

Carbon isotope ratio (*δ* ^13^C) generally tended to increase (i.e., become less negative) for elevated temperature plots, particularly the T_2_ and T_3_ treatments, with a mean *δ* ^13^C for T_2_ and T_3_ of −28.1‰ and −28.0‰ (Fig. 2A). This represented a 0.7‰ enrichment relative to the control plots and a 1.6‰ shift relative to the T_1_ plots. The *δ* ^13^C of T_1_ plots were the least enriched, with mean *δ* ^13^C of −29.6‰. These effects of elevated temperature were also consistent across plant type, with enriched *δ* ^13^C observed under T_2_ and T_3_ for both herbs and grasses alike. Similarly, a lower *δ* ^13^C was observed at T_1_ for both grasses and herbs. In general, *δ* ^13^C appeared to be somewhat more responsive in grasses compared to herbs, with a 1.1‰ shift observed between T_control_ and T_2_/T_3_ plots for grasses, while the shift for herbs was 0.75‰ (Fig. 2B and C).

The impact of heating treatments on the oxygen isotope ratios was more ambiguous. Values of *δ* ^18^O ranged from 22.2‰ for T_1_ to 23.2‰ for T_2_ with large variation within treatments (Fig. 2A). While mean values of *δ* ^18^O appeared to follow a similar pattern to that of *δ* ^13^C (with *δ* ^18^O more enriched in the T_2_ and T_3_ treatments relative to the control and lower *δ* ^18^O observed in the T_1_ treatment compared to the control), there was no significant difference of heating treatment on *δ* ^18^O. Although not significant, there appeared to be different responses in the *δ* ^18^O of grasses and herbs to increased temperature. The *δ* ^18^O of grasses was observed to be more responsive ranging from 21.9 for T_1_ to 23.3 for T_2_ (Fig. 2B). By comparison, the *δ* ^18^O of herbs ranged from 22.4 for T_control_ to 23.0 for T_1_ (Fig. 2C).

### Soil characteristics

Before commencement of the experiment, the soil carbon content was 5.13% (SD 0.57). In 2014, after more than 6 years of ecosystem warming, soil carbon content was in the following order T_1_ < T_3_ < T_control_ < T_2_, with values being, respectively, 17.5, 13.3, 8.0, and 7.0 percent lower than measurements taken before commencement of the experiment. There were no significant differences in the carbon content (%) between the four treatments in 2014 (*P* = 0.168); however, T_1_ had a significantly lower percentage of carbon in 2014 compared to the soil samples taken in 2007 (*P* = 0.012). When comparing the soil samples taken in 2007 around the T_1_ plots to the other samples taken in 2007, no significant differences in the percentage carbon were found. The carbon stocks (kg m^−2^), of the upper 7.5 cm of the soil, were in 2014 in the following order: T_3_ < T_2_ < T_1_ < T_control_, differences, however, were not significant. Values of organic matter content, C content, C/N ratio, and bulk density can be found in Table 5. No significant differences between treatments were found in these soil characteristics, except for the bulk density of the top 7.5 cm, which was significantly higher for T_1_ compared to T_2_ (*P* = 0.023), and tended to be higher for T_1_ compared to T_3_ (*P* = 0.056).

**Table 5:** Average soil characteristics with the standard deviation between brackets, based on samples taken in May 2014. The top layer is the upper 7.5 cm of the soil, the bottom layer is between 15 and 7.5 cm

Soil characteristic	Layer	T_control_	T_1_	T_2_	T_3_
Bulk density (g cm^−3^)	Top	0.92 (0.06)	1.02 (0.06)^a(b)^	0.86 (0.02)^a^	0.86 (0.05)^(b)^
	Bottom	1.06 (0.07)	1.03 (0.14)	0.87 (0.08)	0.96 (0.15)
Organic matter (%)	Top	12.76 (0.53)	10.97 (0.41)	12.60 (1.19)	11.97 (1.20)
	Bottom	10.27 (0.94)	10.87 (0.48)	11.93 (1.26)	10.38 (0.88)
Organic matter (kg m^−2^)	Top	8.77 (0.44)	8.40 (0.76)	8.10 (0.80)	7.79 (1.20)
	Bottom	7.07 (0.78)	8.31 (0.51)	7.67 (0.89)	6.75 (0.87)
Carbon content (%)	Top	4.72 (0.26)	4.23 (0.23)	4.77 (0.33)	4.45 (0.55)
Carbon content (kg m^−2^)	Top	3.25 (0.25)	3.24 (0.28)	3.06 (0.19)	2.90 (0.51)
C/N ratio	Top	13.30 (0.62)	13.06 (0.18)	13.21 (0.81)	12.67 (0.32)

Superscript letter is significant difference between treatments (*P* < 0.05). Superscript letter between brackets is tendency in difference between treatments (*P* < 0.10).

## Discussion

This study showed that even after 6 years of ecosystem warming, NEE was stimulated by increasing soil temperature (at 5 cm depth) by 2 or 3°C. An increase in temperature would thus lead to an increase in loss of C from the system. Based on the modeled data, a soil temperature increase of 2 or 3°C caused an increase in NEE of 1.4 kg CO_2_ m^−2^ year^−1^, the equivalent of 0.38 kg C m^−2^ year^−1^, compared to the control treatment. Although to a lesser extent, analysis of soil C content also confirmed the higher carbon losses from T_2_ and T_3_. At the end of the experiment, carbon stocks in the top 7.5 cm were 0.19–0.35 kg m^−2^ lower compared to the control treatment, which was in line with previous research elevating temperature by 1–3°C (Lu et al. 2013).

When comparing NEE fluxes during day‐ and night‐time, it becomes clear that during night‐time the differences between treatments were more often significant than during daytime. This suggests that during daytime changes in R‐eco are generally offset by counteracting changes in photosynthetic uptake. However, while R‐eco is increased throughout the entire day increased photosynthesis occurred only during daytime and was not enough to counter balance the respiratory losses. Based on the modeled 2013–2014 data, an increase in temperature of 2 or 3°C would lower the carbon storage potential by around 4 tonnes of C ha^−1^ year^−1^.

Generally, temperate grasslands are considered carbon sinks (Jones and Donnelly 2004). However, in the current experiment, a net carbon loss was determined by the modeled NEE, with losses between March 2013 and February 2014 estimated to be between 2.3 and 5.3 kg CO_2_ per m^2^ depending on the treatment. These modeled NEE values were high compared to previous research of comparable unheated grassland sites, in which values ranged between −2.4 and 0.8 kg CO_2_ m^−2^ year^−1^ (Falge et al. 2002; Gilmanov et al. 2007). The metal plates and IR lamps were, because of their height and size, not considered to have a significant shading effect on the plots. However, NEE values, used for modeling, were measured using clear LI‐COR chamber that did have a nontransparent arm and measurement device. This could have caused some shading during NEE measurements. As global radiation was measured by a nearby station, and not within the chamber, this could have caused an overestimation of the NEE. It did however not affect the treatment differences.

Corresponding gross primary productivity (GPP) ranged from 1.8 to 6.8 kg CO_2_ m^−2^ year^−1^ (Gilmanov et al. 2007). The modeled GPP of the current experiment ranged between 2.4 and 3.7 kg CO_2_ m^−2^ year^−1^ depending on treatment and was thus within this range, although on the lower end. Also the maximum daily photosynthetic uptake was within the range found in previous research (Gilmanov et al. 2007). The R‐eco, on the other hand, was higher than previously reported values ranging from 1.8 from 5.5 kg CO_2_ m^−2^ year^−1^ (Gilmanov et al. 2007; Riederer et al. 2015). The control treatment had an R‐eco of 6.3 kg CO_2_ m^−2^ year^−1^, and a soil temperature increase of 2 and 3°C caused a further increase in respiration of 2.7 and 1.4 kg CO_2_ m^−2^ year^−1^, respectively. The high NEE values are therefore mainly caused by high R‐eco values. The organic C content of the current experimental field has generally declined since 1998 (Lenhart et al. 2016). The relatively high R‐eco values could be due to the reduced N fertilization since 1995, leading to an increase in mineralization (Müller et al. 2011). Also land‐use changes before establishment of the permanent grassland site, at least 100 years ago, could lead to high R‐eco values, as soil organic carbon stocks could continue to decline for more than 120 years (Jenkinson and Wild 1988; Smith et al. 2007).

In 2013–2014, the lowest values of R‐eco, NEE, and biomass yield were unexpectedly found for treatment T_1_. Even though in the first three years of the experiment, R‐eco was, as expected, in the following order T_control_ < T_1_ < T_2_ < T_3_. When comparing the *δ* ^13^C/*δ* ^18^O correlations of bulk leaf material, using a concept proposed by Scheidegger et al. (2000) to determine the treatment effect on stomatal conductance and photosynthesis, the negative correlation at T_1_ for both the herbs and grasses indicated a static photosynthetic capacity and increased leaf conductance or even reduced photosynthesis. This was determined via changes in both *δ* ^13^C and *δ* ^18^O, reflecting changes in both the substomatal CO_2_ concentration and evaporative demand during the entire period during which organic matter was produced (Gillon et al. 1998; Ehleringer and Sandquist 2006). The *δ* ^13^C signature of C‐3 plants primarily reflects isotopic fractionation associated with the enzyme RUBISCO (Farquhar et al. 1982; Lanigan et al. 2008), while *δ* ^18^O increases with increased evapotranspiration (Saurer et al. 1997). The lowest aboveground biomass production found under T_1_, also reflects a reduction in photosynthesis. Previous research suggested that increased soil respiration due to ecosystem warming would only be short‐lived until the most labile soil carbon was depleted (Bradford et al. 2008). When substrate is abundant, and soil moisture is sufficient, the respiration rate will be governed by temperature changes. However, when substrate is limited an increase in temperature could lead to relatively lower increases in respiration (Davidson and Janssens 2006). Reduced photosynthesis would cause a decrease in labile organic carbon input. It is therefore possible that the reduced NEE and R‐eco from T_1_ in the final year of the experiment could be caused by a depletion of labile carbon, due to reduced C inputs in the previous years.

Increases in temperature would not indefinitely increase photosynthesis, as photosynthesis has an optimal temperature within the range of temperatures regularly observed, and increases in temperature would also lead to decreased soil moisture, which could adversely affect plant growth (Luo 2007). In the current experiment, temperature was altered using IR heaters. These heaters will not only increase temperature, but also decrease soil moisture via a temperature effect on transpiration (Peng et al. 2014). Boeck et al. (2012) calculated that a 1° increase in temperature would cause a 12–15% increase in transpiration; however, changes in relative humidity would significantly alter this percentage. In the current experiment, aboveground biomass was lower under T_3_ than under T_2_, although not significantly. It has to be noted that the plot size, due to the locality of the IR lamps, had to be small (317.8 cm^2^), possibly resulting in less significant differences. This reduction in biomass production in T_3_ is possibly related to the temperature effect on photosynthesis in this treatment which was arguably above the optimal temperature for photosynthesis or due to a constrain imposed by the decreasing soil moisture. Surprisingly, the shift in *δ* ^18^O under the high‐temperature treatments (T_2_ and T_3_) was low, indicating that increased evapotranspirative demand at higher temperatures may not have been that high, or was variable. And the *δ* ^13^C/*δ* ^18^O correlations indicated no major implication to photosynthesis in T_3_ compared to T_2_. Leaf material was collected after the first growth period (in May, corresponding to the soil sampling). Possibly an increased drought stress in T_3_, leading to a reduction in respiration, is only prevalent in the summer, which is also supported by the NEE data showing a decrease in T_3_ compared to T_2_ in the summer months.

The importance of including soil moisture data in modeling CO_2_ fluxes became apparent in this study. The *R* ^2^ of the R‐eco model changed from 0.68 to 0.78 when a soil moisture component was added to the model. The lower *R* ^2^ without addition of a soil moisture component was associated to an overestimation of R‐eco during a period of drought, in August 2013. Under reduced soil moisture, the diffusion of extracellular enzymes and soluble organic carbon substrates is reduced, lowering the substrate availability to reaction microsites (Davidson and Janssens 2006). Future changes in the weather could significantly alter not only temperature but also soil moisture because of changes in precipitation patterns and amounts. It is therefore important to include changes in soil moisture due to changes in precipitation in future predications of CO_2_ emissions.

In conclusion, a net soil carbon loss was observed under all treatments. But the continuing onset of climate change could lead to further significantly increased carbon losses from temperate grasslands. The increased losses were predominantly caused by an increase in respiration rather than a reduced photosynthetic activity. Thus, in the current experiment, these increases could not be offset by increased photosynthesis, although NEE during daytime was very similar for all treatments. To improve prediction of the extent of carbon losses in the future, it is important to not only look at changes in temperature but also include changes in precipitation with subsequent changes in soil moisture and the combined effects this has on ecosystems C dynamics.

## Conflict of Interest

None declared.
